# Does having children increase environmental concern? Testing parenthood effects with longitudinal data from the New Zealand Attitudes and Values Study

**DOI:** 10.1371/journal.pone.0230361

**Published:** 2020-03-18

**Authors:** Taciano L. Milfont, Wouter Poortinga, Chris G. Sibley

**Affiliations:** 1 School of Psychology, Victoria University of Wellington, Wellington, New Zealand; 2 School of Psychology, Cardiff University, Cardiff, United Kingdom; 3 School of Psychology, University of Auckland, Auckland, New Zealand; Tilburg University, NETHERLANDS

## Abstract

Having children is a transformative experience and may change the way people think about the future. Parents invest time, energy and resources to ensure the survival and reproductive success of offspring. Having children may also induce environmental concerns and investments in actions aimed at guaranteeing the quality of natural resources available to offspring. However, there is limited empirical support for this parenthood effect, and little is known about how environmental attitudes and behaviour change over time following the birth of a child. This pre-registered study uses data from the first seven waves (2009–2015) of the New Zealand Attitudes and Values Study—a longitudinal national probability study of social attitudes, personality, and health outcomes—with multilevel interrupted time series analysis. Respondents’ belief in the reality and causes of climate change, sacrifices to standard of living to protect the environment, and changes in daily routine to protect the environment did not change significantly following the birth of a child; and nor were there changes in the underlying trends of attitudes or pre-birth anticipation effects. The study further found no gender differences in the attitudinal effects of childbirth. Additional exploratory analyses suggest that becoming a parent for the first time may increase beliefs in the reality of climate change but does not appear to change other environmental attitudes. Overall, our findings provide little empirical evidence for parenthood effects on environmentalism.

## Introduction

Parenting takes time, energy and resources but pays off evolutionarily because parental investment increases the survival and reproductive success of offspring to which parents are genetically related. Since the survival and reproductive success of offspring is dependent on the availability and quality of natural resources, it is reasonable to expect that parental investment in offspring must also incur considerations about the future of such resources. Support for this expectation emerges from findings indicating that parenthood may increase environmental concern, particularly for women (e.g., “Motherhood Effect”, [1]; “Parental Roles Hypothesis”, [2]; “Parenthood Status Hypothesis”, [3]). Although having children might not affect personality trait development [e.g., 4, 5], it influences relationship quality and marital satisfaction [e.g., 6, 7] and having children may influences other aspects of people’s lives. Importantly, parents have feelings of stewardship, social responsibility and affinity to offspring and are thus more likely to consider conditions affecting offspring in the future. In this article, we used seven waves of longitudinal data to investigate the direct effect of childbirth on parents’ environmental attitudes and behaviours. Although our focus is on possible direct parenthood effects, it is important to note factors that might explain such effects on environmentalism.

Many factors change as a result of childbirth, which eventually might affect environmentalism. Parenthood has been associated with stress, income changes, gender-specific division of labour [e.g., 8–10]. Because parents must dedicate time, resources and energy for the immediate health and wellbeing of offspring, such changes and pressing issues might have detrimental effects on environmentalism. For example, time pressure due to childcare activities and responsibilities [11] could reduce parents’ motivation or ability to act pro-environmentally, and per capita environmental footprint also increases with family size [12]. These considerations indicate that parenthood might have an indirect and negative impact of environmentalism (e.g., childbirth → time pressure → reduced environmentalism). Although this negative parenthood effect is plausible, there is more theoretical and preliminary empirical evidence of a positive parenthood effect, particularly from the literature discussing future thinking and legacy motives (e.g., childbirth → legacy motivation → increased environmentalism).

In their analyses of intergenerational decisions and legacy motivations, Wade-Benzoni and colleagues defined legacies as enduring meaning connected to the identity of an individual actor and manifested in actions intended to have an impact beyond the temporal constraints of the lifespan of the actor [13, 14]. More recently, Wade-Benzoni [15] argued that a legacy “is only meaningful and emergent in a context where a person’s behaviour has implications for *other* people in the *future*” (p. 19; emphasis in the original).

These conceptual analyses of legacy do not explicitly consider leaving a positive legacy regarding the natural environmental, but empirical studies have linked domain-general legacy motives and environmental protection. Milfont and Sibley [16] examined the associations between a measure of generativity—a concern for forming and guiding the next generation [17]; e.g., “I try to pass along the knowledge I have gained through my experiences”, “I think that I will be remembered for a long time after I die”—and both pro-environmental attitudes and retrospective self-reports of ecological behaviours. Regression results showed that individuals who reported greater levels of generativity had greater preservation attitudes and ecological behaviours, and this association held even after controlling for their future orientation and other-focused values.

Using a conceptually related measure of legacy (e.g., “I have important skills I can pass along to others”, “I care about what future generations think of me”), Zaval, Markowitz and Weber [18] showed that higher levels of legacy motives were associated with higher pro-environmental beliefs and intentions, and also greater donation behaviour to an environmental non-profit organisation. Zaval and colleagues extended these correlational findings in a follow-up experiment and showed that participants in the legacy-motive-inducing prime showed greater pro-environmental intentions and donation behaviour than those in the control condition. This experimental effect is conceptually similar to findings from a previous experimental study showing that priming participants to envision their everyday life in the future increased environmental concern compared to those primed to think about their current everyday life [19].

In conjunction, these findings indicate that individuals who more strongly consider the implications of their behaviour for other people in the future (as indexed by legacy or generativity concerns) are more likely to display pro-environmental engagement. Additionally, these findings are related to research showing that greater levels of pro-environmental engagement is observed for those with higher consideration of the future consequences of their behaviour [e.g., 20] and higher endorsement of other-focused personal values [e.g., 21], as well as to research showing that legacy motives are linked to feelings of stewardship, social responsibility concerns, and affinity to future generations in intergenerational decision making [for a review, see 15].

A recent longitudinal study has explicitly tested whether having children impacted the environmental attitudes and behaviours of parents. Thomas and colleagues [22] used data from the Understanding Society Survey, a longitudinal dataset representative of the UK population (*N* = 18,176), to test four predictions: (1) whether having a child changes people’s environmental attitudes and behaviour, irrespective of whether the child was a firstborn or not; (2) whether having a firstborn (i.e., becoming a parent for the first time) changes people’s environmental attitudes and behaviour; (3) whether any changes in attitudes and behaviour are restricted to new parents with high levels of environmental concern; and (4) whether any changes in attitudes and behaviour, are evident in both new mothers and fathers.

The findings did not support their predictions, as having a new child was associated with a very small *decrease* in the frequency of environmental behaviours; moreover, only parents with already high environmental concern showed a small increase in the *desire* to act more sustainably after the birth of their first child. Overall, the findings observed by Thomas and colleagues [22] tend to support findings showing that per capita environmental footprint increases with family size [e.g., 23], indicating that parenthood might have a negative (rather than positive) effect on environmental protection due to family pressures (e.g., need to have warmer / cooler homes, increase travels and purchases), at least soon after parenthood.

### Aims of this study

The goal of the present study was to further test the likely of parenthood effects on environmentalism using longitudinal data from the New Zealand Attitudes and Values Study (NZAVS; www.nzvalues.org)—a longitudinal national probability study of social attitudes, personality, and health outcomes. Over the course of the study, the NZAVS has randomly sampled more than 20,000 New Zealanders over seven waves of data collection and followed the same group of roughly 15,000 each year from 2009–2015.

The main analysis of the present study was pre-registered with the Open Science Framework (https://osf.io/uzbxk/), complemented with an additional exploratory analysis described below. The first pre-registered aim of the study (Aim 1) involved testing whether having a child changes people’s attitudes towards the environment. It is also possible that the experience of having a child, and its potential influence on environmental attitudes and behaviour, might be different for mothers and fathers. To test this possibility, and following the approach taken by Thomas and colleagues [22], we examined whether there are gender differences in the attitudinal effects of childbirth (Aim 2). This pre-registered analysis was to test whether the predicted parenthood effect applies to both men and women. The third aim of the study was not pre-registered but suggested by the reviewers of the manuscript. This explored whether the attitudinal effects of childbirth are different for existing as compared to new parents (Aim 3). That is, a firstborn may have a different impact on parents’ environmental attitudes than a nextborn has. As the third aim was not pre-registered, these analyses are deemed exploratory.

Thomas and colleagues [22] were constrained by the environmental questions included in the longitudinal dataset, and the present study is also constrained by the data structure and the environmental questions asked in the NZAVS dataset. However, the NZAVS dataset affords testing parenthood effects in relation to three environmental domains: climate change beliefs, pro-environmental intentions, and retrospective self-reports of pro-environmental behaviour. In addition, and in contrast to analysis by Thomas et al. which was constrained to two time points only, the NZAVS dataset included the different environmental questions in five to seven waves, and thus affords multiple measurements before and after the birth of a child. This means that the NZAVs is not only able to detect sudden ‘discontinuous’ mean-level changes from before to after childbirth but could potentially show gradual slope-level changes in environmental attitudes in anticipation of and following childbirth. These effects can only be detected with datasets that have multiple pre- and post-event measures.

**Fig 1:**
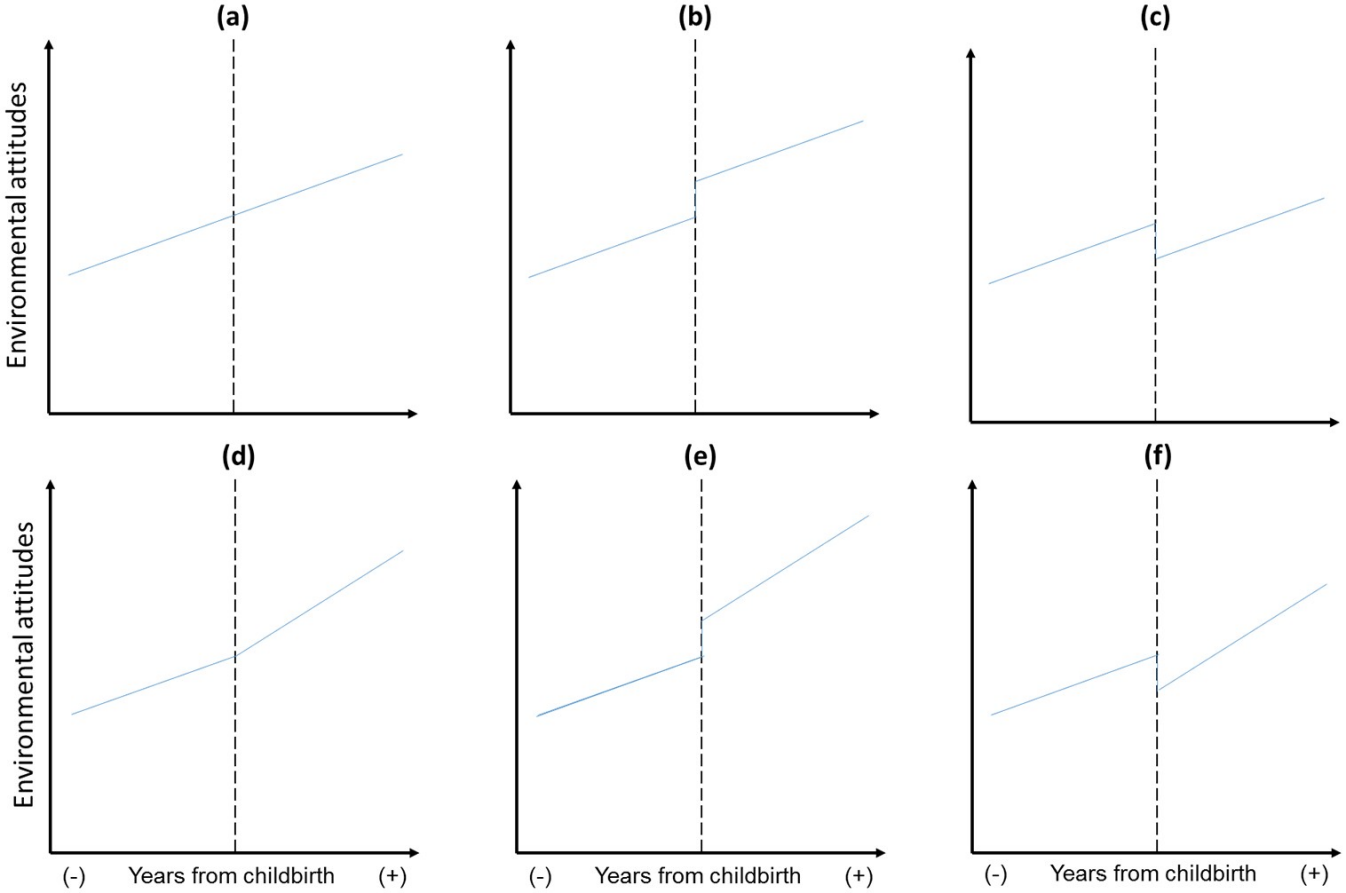
Six different patterns of change in environmental attitudes from before to after childbirth.

Life events, such as childbirth, can have abrupt effects and lead to more gradual changes over time [24]. Fig 1 presents an overview of a number of possible patterns of change from before to after childbirth. Fig 1a shows a pattern where there is no change in environmental attitudes as a result of childbirth that is distinct from an underlying (upward) secular trend. Fig 1b illustrates a level-change model depicting the main prediction of parenthood effects that the birth of a new child *increases* pro-environmental intentions/behaviour. Fig 1c illustrates a different impact model of becoming a parent, with a *decrease* in pro-environmental intention/behaviour because of contextual demands of parenthood. This level change model is related to findings reported by Thomas and colleagues [22], indicating that families are less likely to perform pro-environmental behaviours because of family pressures. There are also other possible effects following birth of a child, in that there may be a change in the underlying trend (as indicated by a change in slope) rather than a discontinuous trajectory (see Fig 1d). It is possible that childbirth has an effect of decelerating or accelerating environmental attitudes over time. And then finally, there could be combinations of these different effects as shown in Fig 1e and 1f.

There are also other effects possible, in that there may be lags in possible mean-level and slope-level changes (for other examples see [25]). For example, an initial decrease in pro-environmental attitudes and behaviour in the early stages of parenthood, due to the demands of looking after a young child, may be followed by an increase in pro-environmental attitudes and behaviour in later stages of parenthood to ensure an environmental legacy for offspring. In addition to changes that follow childbirth, there may be anticipatory effects pre-birth. Any event that is anticipated, including childbirth, can produce effects in preparation for the event itself [24]. It may be that prospective parents already start considering environmental issues before the arrival of the child.

Our analyses focused on confirmatory tests to identify sudden and gradual effects following childbirth. In operational form, we tested a level change model whereby parenthood leads to an immediate change in the level of respondents’ pro-environmental intentions/behaviour when compared to levels prior to the birth of a new child, and for possible changes in the underlying trends of the respondents’ pro-environmental intentions/behaviour from before to after the birth of a child. The analysis controlled for anticipatory effects to avoid the results being biased by possible pre-event changes. The anticipatory controls were not pre-registered but suggested by the reviewers of the manuscript. A number of pre-birth anticipatory effects have been reported in the literature, such as changes in health-related behaviours [26] and self-control [27]. Scholars recommend considering anticipatory effects when examining life-event changes as ignoring them may bias the results [24]. It is for example possible that (positive) pre-event changes obscure real effects. This may then lead to a false conclusion that there is no change or a negative change associated with becoming a parent.

## Method

### The New Zealand Attitudes and Values Study

#### The study

The New Zealand Attitudes and Values Study (NZAVS) is a nation-wide study that has been assessing the socio-political attitudes of New Zealanders from 2009 onwards. People randomly sampled from the New Zealand Electoral Roll received invitations to participate in a mail-based survey. They were posted a copy of the survey, along with a second postal follow-up two months later. Those who provided an email address were also emailed and invited to complete an online version instead (for more information, see www.nzvalues.org).

#### Ethics information and data availability

The NZAVS is reviewed every three years by the University of Auckland Human Participants Ethics Committee. The first phases of the longitudinal study were approved on 09 September 2009 for three years (reference number: 2009/336). Ethics approval for the study was re-approved by the University of Auckland Human Participants Ethics Committee on 17 February 2012 until 09 September 2015 (reference number: 6171), and then on 03 June 2015 until 03June 2018 (reference number: 014889). All participants granted informed written consent. Contact details are removed when the questionnaires are received, and all data were de-identified before analyses were conducted. NZAVS data is hosted at the University of Auckland, New Zealand. Data cannot be made available due to ethical restrictions imposed by the Ethics Committee. A de-identified dataset is available upon request from the corresponding author for all appropriately qualified researchers. More information regarding requests for data access may be found here: https://www.auckland.ac.nz/en/about/research/re-ethics/re-uahpec.html.

#### Participants

The longitudinal dataset used for the analyses was created by combining the first seven waves of the NZAVS, covering the period from 2009 to 2015. Wave 1 (2009) of the NZAVS contained 6,518 participants. Wave 2 (2010) retained 4,423 participants who also took part in the first wave of the survey. Each subsequent survey contacted all participants who took part in the previous wave, with new additions from booster sampling. Wave 3 (2011) was boosted through a survey posted on the website of a major New Zeeland newspaper and included responses from 6,884 participants (of 3,918 were retained from previous waves). Wave 4 (2014) contained responses from 12,182 participants, of which 6,807 could be linked to individuals who took part in one of the previous waves. Five booster samples in Wave 4 consisted of a random national sample from the New Zealand Electoral Roll, a random regional sample of people living in the Auckland area, a random regional sample of people living in the Christchurch area, a sample of individuals living in areas of moderate-to-high deprivations from across the country, and a random national sample of individuals that were of Māori ethnicity. Wave 5 (2013) had a final sample of 18,264 participants, of which 10,502 were retained from the previous surveys, and two booster samples were conducted to recruit new participants. The first booster sample targeted people who were between 18 and 60 years old, and recruited 7,489 participants. The second booster sample targeted those who were of Māori ancestry and recruited 92 participants. Wave 6 (2014) contained responses from 15,822 participants, of which 14,875 could be matched to one of the previous surveys, and no booster sample was collected. Wave 7 (2015) included responses from 13,944 participants of which 13,879 could be matched to one of the previous surveys, and no booster sample was collected.

The resulting dataset consisted of 78,037 observations within 23,027 unique individuals, meaning an average of 3.4 observations per individual across the seven waves. All multiple observations were recorded in contiguous waves. Most of the respondents were female (61.9%) and classified as New Zealand European (83.5%). The average age of respondents at the time of Wave 1 was 43.5 years old (*SD* = 14.7). Table 1 further shows that there were 1,522 newborns across the seven waves, of which 1,104 were nextborns.

**Table 1:** Characteristics of the seven waves of New Zealand Attitudes and Values Study.

	Wave 1 (n = 6,518)	Wave 2 (n = 4,423)	Wave 3 (n = 6,884)	Wave 4 (n = 12,182)	Wave 5 (n = 18,264)	Wave 6 (n = 15,822)	Wave 7 (n = 13,944)	Overall (n = 23,027)
**Gender**								
Female	59.5%	61.6%	62.5%	62.6%	62.7%	63.2%	62.6%	61.9%
Male	40.5%	38.4%	37.4%	37.2%	37.2%	36.6%	37.2%	37.8%
**Age**								
Mean (SD)	48.0 (15.8)	51.0 (15.2)	50.6 (15.9)	49.1 (15.0)	47.7 (14.1)	49.3 (14.0)	50.8 (13.9)	43.5 (14.7)^(1)^
**Ethnicity**								
New Zealand European	81.9%	85.9%	74.8%	84.4%	85.5%	89.5%	89.7%	83.5%
**New Zealand Deprivation Index**								
Q1	26.1%	27.2%	25.9%	25.4%	25.7%	25.4%	26.0%	25.5%
Q2	21.9%	21.7%	21.7%	22.5%	22.8%	22.9%	22.7%	21.5%
Q3	19.9%	19.2%	20.0%	19.6%	19.5%	19.6%	19.4%	19.7%
Q4	17.6%	17.3%	17.8%	17.7%	17.5%	17.4%	17.3%	17.6%
Q5	14.5%	14.6%	14.7%	14.7%	14.5%	14.7%	14.5%	14.5%
Mean (SD)	4.93 (2.83)	4.88 (2.86)	4.95 (2.84)	4.95 (2.83)	4.92 (2.82)	4.93 (2.83)	4.90 (2.83)	4.95 (2.83)
**Number of children**								
0	25.1%	20.6%	24.4%	22.6%	24.0%	25.0%	24.0%	—
1	11.6%	11.0%	10.2%	11.5%	11.8%	11.6%	11.8%	—
2	28.7%	31.1%	29.6%	29.5%	30.3%	31.6%	32.7%	—
3 or more	34.5%	35.8%	32.4%	31.7%	29.3%	30.1%	30.3%	—
**Number of newborn**	—	231	129	170	262	419	311	1,522
**Number of nextborn**	—	159	103	126	197	305	214	1,104

The numbers in the table may not always add up to 100% due to missing values;

^(1)^ Age was standardised to the time of Wave 1. Q = quartile.

## Measures

### Independent variables

The independent variable for the study was thus newborn status during the study period. This *newborn status* variable was established by comparing the number of children reported in the different waves of the NZAVS (“How many children have you given birth to, fathered, or adopted?”). The variable was coded 0 before the birth of a child, and 1 after the birth of a child. The variable remained coded as 0 if individuals did not have a new child during the period of the study. We further established whether participants already had children at the time of the birth of a new child. This was determined by the same number of children variable. An individual-level dummy variable was created indicating whether participants had already given birth to, fathered, or adopted a child (1) or not (0) before the birth of the new child. This was combined with the newborn status variable to create a *nextborn status* variable. In addition, we created an interaction term between *gender* and *newborn status* to explore differences between new mothers and new fathers.

### Dependent variables

Six dependent variables were considered covering people’s beliefs about climate change and their willingness to make changes for the environment. First, respondents were asked to indicate to what extent they agree with the two “*Climate change belief*” statements: “Climate change is real” and “climate change is caused by humans”. They could respond using a 5-point scale ranging from 1 (*strongly disagree*) to 7 (*strongly agree*). The questions were asked in all seven waves of the NZAVS. Second, respondents were asked (a) whether they *are willing* to make sacrifices to their standard of living (e.g., accept higher prices, drive less, conserve energy), and (b) whether they *have made* sacrifices to their standard of living in order to protect the environment. In both cases they could respond using a 7-point scale ranging from 1 (*definitely yes*) to 7 (*definitely no*). These questions were asked from Wave 1 to Wave 6 of the NZAVS, and referred to as “*Sacrifices to standard of living to protect the environment*”. Third, respondents were asked (a) whether they *are willing* to change their daily routine, and (b) whether they *have made* changes to their daily routine in order to protect the environment, answered the same 7-point scale ranging from 1 (*definitely yes*) to 7 (*definitely no*). These “*Changes in daily routine to protect the environment*” questions were included in Wave 1 to Wave 5 of the NZAVS. All dependent variables were standardised as Z scores.

### Control and additional variables

Four control variables were included in the analyses: *gender* (1 = male, 0 = female), *age* (in years), *ethnicity* (1 = New Zealand European, 0 = other), and *neighbourhood deprivation*. Neighbourhood deprivation was measured using the 2013 New Zealand Deprivation Index [28]. The New Zealand Deprivation Index ranges from 1 (*least deprived*) to 10 (*most deprived*). Other variables included in the interrupted time series analysis were the year of the survey (*survey year*; ranging from 1 = 2009 to 7 = 2015), time elapsed since having the child (*newborn year*), and the time leading up to having a new child (*anticipation year*). The *newborn year* variable was coded as 0 in all years before the event and as incremental years following the event. The anticipation year variable was coded as negative years preceding the event, and as 0 in all years following the event. The two variables were coded 0 throughout if there was no birth in the period of the study.

### Statistical analysis

The data were analysed from a multilevel interrupted time series perspective using the MLwiN 2.36 software package [29]. The interrupted time series study design is increasingly being employed to evaluate public health interventions because it is a useful quasi-experimental design to evaluate the longitudinal effects of interventions with regression models, and particular useful when evaluating “natural experiments” in real-world settings [see 30]. Given a continuous sequence of repeated observations of a particular outcome of interest measured over time (or time series), this analytical approach examines whether an event (in this case child birth) changes the mean or underlying over-time trend of a particular outcome. In this study, the outcomes of interest are observations of the six dependent variables across the seven waves, and the ‘interruption’ is the birth of a new child.

Randomisation of population members becoming a parent is not viable, so we have a quasi-experimental design or “natural experiment” occuring in an real-world settting. Hence, the time series analysis allows us to test statistically whether becoming a parent interrupts the underlying over-time trend in the six dependent variables. We employed a multilevel approach because 78,037 observations are nested within the 23,027 unique individuals that took part in the study. This nested design allows the time dependent nature of the data to be taken into account. A power calculation suggests that the dataset has a statistical power of 0.95 at the 5% significance level for the main analysis to detect small effect sizes of approximately 0.08. This calculation considers the design effect of employing a two-level structuring of the data [31, 32].

The main pre-registered analysis consisted of a series of multilevel models (Models 1) to address the first aim of the study (Aim 1). This analysis included *newborn status* at the seven time points as the independent variable, and the six variables assessing climate change beliefs, environmental attitudes, intentions and past behaviour at the same time points as the dependent variables. The analysis shows whether there is change in environmental engagement from before to after having a new child during the period of the study (as indicated by *newborn status*), while controlling for any underlying trend in the dependent variables across time (as indicated by *survey year*). The variable indicating the time elapsed since having the child (*newborn year*) shows whether there is a change in the underlying trend from before to after having a child. The variable indicating the time leading up to having a new child (*anticipation year*) shows whether there is a change in the underlying trend from before to after having a child. The *anticipation year* variable was not mentioned in the pre-registration but included on the suggestion of reviewers to control for any pre-event changes that could bias the results.

We further examined whether there are differences in the attitudinal effects of childbirth between men and women (Aim 2). This analysis was also pre-registered and was conducted by adding a *Gender* x *Newborn Status* interaction term, indicating whether attitudinal effects of childbirth are different for men as compared to women (Models 2).

The third, exploratory, analyses were conducted to examine whether there are differences in the attitudinal effects of childbirth for firstborns and nextborns; or, in other words, whether any changes in attitudes only happen for new parents as compared to existing parents (Aim 3). This was done by adding the *nextborn status* variable, indicating whether the attitudinal effects of childbirth are different for existing as compared to new parents (Models 3).

In all models, gender, age, ethnicity and neighbourhood deprivation were included as covariates at the individual level, which means that these control variables were considered invariant over time. Age was standardised to 2009; that is, the age of participants at the time of the first wave of the study. As noted, the analyses further included a variable indicating the year of the survey and a variable indicating the time elapsed since having a child during the period of the study.

## Results

### Childbirth and environmental engagement

Table 2 presents the results of the first six interrupted time series regressions to examine whether having a child changes people’s pro-environmental tendency (Aim 1). The output from MLwiN for these and all following analyses is given in the Supporting Information document available online.

**Table 2:** Results of the multilevel interrupted time series analyses testing the environmental legacy hypothesis (Models 1).

	Beliefs about climate change		Sacrifices to standard of living to protect the environment		Changes in daily routine to protect the environment	
	Climate change is real	Climate change is caused by humans	Willing to make	Have Made	Willing to make	Have Made
Predictor	B (95% CI)	B (95% CI)	B (95% CI)	B (95% CI)	B (95% CI)	B (95% CI)
Newborn Status	-0.003 (-0.064 to 0.058) ^n.s.^	0.052 (-0.060 to 0.164) ^n.s.^	-0.037 (-0.111 to 0.037) ^n.s.^	0.001 (-0.075 to 0.077) ^n.s.^	0.095 (-0.007 to 0.197) ^n.s.^	0.048 (-0.054 to 0.150) ^n.s.^
Newborn Year	-0.008 (-0.032 to 0.016) ^n.s.^	0.005 (0.001 to 0.009) ^n.s.^	0.002 (-0.029 to 0.033) ^n.s.^	0.008 (-0.023 to 0.039) ^n.s.^	-0.017 (-0.066 to 0.032) ^n.s.^	-0.004 (-0.053 to 0.045) ^n.s.^
Anticipation Year	0.003 (-0.015 to 0.021) ^n.s.^	-0.003 (-0.021 to 0.015) ^n.s.^	0.017 (-0.001 to 0.035) ^n.s.^	-0.007 (-0.025 to 0.011) ^n.s.^	-0.004 (-0.022 to 0.014) n.s.	-0.021 (-0.039 to -0.003)*
Survey Year	0.056 (0.052 to 0.060)***	0.063 (0.059 to 0.067)***	0.021 (0.015 to 0.027)***	0.000 (-0.006 to 0.006) ^n.s.^	0.056 (0.050 to 0.062)***	0.056 (0.050 to 0.062)***
Gender (1 = male, 0 = female)	-0.12 (-0.134 to -0.106)***	-0.172 (-0.186 to -0.158)***	-0.215 (-0.233 to -0.197)***	-0.234 (-0.250 to -0.218)***	-0.203 (-0.223 to -0.183)***	-0.272 (-0.292 to -0.252)***
Age	-0.062 (-0.068 to -0.056)***	-0.084 (-0.088 to -0.080)***	-0.002 (-0.008 to 0.004) ^n.s.^	0.015 (0.009 to 0.021)***	-0.002 (-0.008 to 0.004) ^n.s.^	0.034 (0.028 to 0.040)***
Ethnicity (1 = New Zealand European, 0 = other)	-0.197 (-0.219 to -0.175)***	-0.25 (-0.272 to -0.228)***	-0.042 (-0.067 to -0.017)**	-0.034 (-0.058 to -0.010)**	-0.104 (-0.131 to -0.077)***	0.014 (-0.013 to 0.041) ^n.s.^
Deprivation	0.012 (0.010 to 0.014)***	0.010 (0.008 to 0.012)***	-0.006 (-0.008 to -0.004)***	0.003 (0.001 to 0.005)**	-0.002 (-0.006 to 0.002) ^n.s.^	-0.002 (-0.006 to 0.002) ^n.s.^

*p < .05.

**p < .01.

***p < .001.

n.s. = statistically non-significant.

The first column of Table 2 shows that the birth of a new child during the time of the study was not associated with the belief that climate change is real. The second column shows that the level in beliefs that climate change is caused by humans also did not change from before to after the birth of a new child during the period of the study. While there was an increase in agreement that climate change is real and caused by humans over time [see also 33], we did not find evidence that there is a change in this trend from before to after the birth of a child. In addition, no pre-birth anticipation effects were found for the two climate change belief variables. Both climate change beliefs were also strongly associated with the covariates. Men were generally less likely to agree that climate change is real or caused by humans, as were older individuals, those of European descent, and those from less deprived neighbourhoods [see also 34].

The third and fourth column of Table 2 present the results for questions asking respondents whether they were willing to make or had made sacrifices to their standard of living in order to protect the environment. The results show that there were no changes in the level and trends associated with the birth of a new child during the period of the study; and there were no changes in the period running up to childbirth. While respondents became increasingly more willing to make sacrifices to their standard of living in order to protect the environment over the period of the study, there was no change in people having actually made such sacrifices. In general, men and those of European descent were less willing to make, or indicated that they had made, sacrifices to their standard of living in order to protect the environment. While respondents living in more deprived neighbourhoods were less willing to make sacrifices, they were more likely to indicate that they actually had made such sacrifices. These effects were small, however.

The fifth and sixth column of Table 2 present the results for questions asking respondents whether they were willing to make or had made changes to their daily routine in order to protect the environment. Again, there were no changes in the level and trends for the questions from before to after the birth of a new child during the period of the study. While there were no pre-birth anticipation effects for people’s willingness to make changes to their daily routine, there was a small negative effect in terms of their self-reports of having made changes. The underlying trend indicates that, over time, people have become more willing to make, or indicated that they had made, changes to their daily routine in order to protect the environment; however, there was no change to these underlying trends from before to after the birth of a child. Similar to findings for the standard-of-living-sacrifice questions, men and those of European descent were less willing to make changes to their daily routine in order to protect the environment. Men and younger participants were less likely to indicate that they had made changes to their daily routine in order to protect the environment. Neighbourhood deprivation was unrelated to either dependent variable. These analyses show that there are no mean-level or slope-level effects following childbirth for parents overall.

### Gender differences

Table 3 presents the results of six interrupted time series regressions (Models 2) to examine whether there are gender differences in the attitudinal effects of childbirth (Aim 2). The models included an interaction term between participants’ gender and having a new child during the period of the study. When added to the model, the interaction term was non-significant for five out of six dependent variables. There was a small-sized effect for having made changes to daily routines to protect the environment, although this effect became non-significant after correcting for multiple comparisons. These analyses show that there are no mean-level or slope-level effects following childbirth for either mothers or fathers.

**Table 3:** Results of the multilevel interrupted time series analyses testing the environmental legacy hypothesis (Models 2 and 3).

	Beliefs about climate change		Sacrifices to standard of living to protect the environment		Changes in daily routine to protect the environment	
	Climate change is real	Climate change is caused by humans	Willing to make	Have Made	Willing to make	Have Made
Predictor	B (95% CI)	B (95% CI)	B (95% CI)	B (95% CI)	B (95% CI)	B (95% CI)
**Models 2**						
Gender x newborn status	-0.062 (-0.127 to 0.003) ^n.s.^	0.064 (-0.065 to 0.193) ^n.s.^	0.027 (-0.051 to 0.105) ^n.s.^	0.038 (-0.040 to 0.116) ^n.s.^	0.024 (-0.076 to 0.124) ^n.s.^	0.115 (0.015 to 0.215)*
**Models 3**						
Newborn status	0.092 (0.012 to 0.172)*	0.053 (-0.061 to 0.167) ^n.s.^	0.003 (-0.097 to 0.103) ^n.s.^	0.049 (-0.051 to 0.149) ^n.s.^	0.117 (-0.014 to 0.248) ^n.s.^	0.009 (-0.124 to 0.142) ^n.s.^
Nextborn status	-0.138 (-0.211 to -0.065)***	-0.045 (-0.131 to 0.041) ^n.s.^	-0.070 (-0.156 to 0.016) ^n.s.^	-0.087 (-0.175 to 0.001) ^n.s.^	-0.051 (-0.161 to 0.059) ^n.s.^	0.025 (-0.087 to 0.137) ^n.s.^

*p < .05.

**p < .01.

***p < .001.

n.s. = statistically non-significant.

### Firstborns versus nextborns

Table 3 further presents the results of six interrupted time series regressions (Models 3) that were constructed to examine whether the attitudinal effects of childbirth are different for existing as compared to new parents (Aim 3). The models included the *nextborn status* in addition to the *newborn status* variable. The newborn status parameter was significant at the p<0.01 level, which in this model indicates that new parents increase the belief that climate change is real after the birth of their first child. In addition, the nextborn status parameter was significant at the p<0.001 level for the belief that climate change is real, which in this model indicates that the effect for existing parents is lower in comparison to the one for new parents. However, correcting for multiple comparison (of 12 parameter estimates) renders both effects statistically non-significant. These additional exploratory analyses provide indications that becoming a parent for the first time *may* increase beliefs in the reality of climate change but does not change any other environmental attitudes.

## Discussion

Past correlational research has demonstrated that greater levels of pro-environmental engagement is associated with generativity and legacy concerns [16, 18], and higher levels of future thinking and endorsement of other-focused personal values [20, 21]. Experimental studies have also shown that priming individuals to envision their everyday life in the future [19], or to describe what they want to be remembered for by future generations [18] led to an increase in pro-environmental engagement. One logical extension of these findings showing an effect of other-focus and future-focus on environmental protection is to examine whether becoming a parent would influence one’s pro-environmental engagement. Parental investment in offspring should include considerations of the availability and quality of the natural environmental necessary for the survival and reproductive success of offspring.

Despite the theoretical and intuitive appeal of parenthood effects on environmentalism, a recent longitudinal study testing whether parenthood would increase pro-environmental engagement did not provide empirical support [22]. In the present study, we employed a distinct longitudinal dataset to test the hypothesis over up to six years. Across six dependent variables, we did not observe a single significant attitudinal effect related to the birth of a child. That is, we did not find any change in pro-environmental tendencies from before to after the birth of a child, and there were no changes in the underlying trends in pro-environmental tendencies either. In addition, the study found no gender differences in the attitudinal effects of childbirth. That is, null results in mean-level and slope-level effects were found for both mothers and fathers. Additional exploratory analyses suggest that becoming a parent for the first time may increase beliefs in the reality of climate change, but no effects were found for the other five environmental measures; and these effects for new parents must be interpreted with caution, as they were rendered non-significant when correcting for multiple comparisons.

Our findings provide little empirical evidence for parenthood effects on environmentalism, supporting the findings observed by Thomas and colleagues [22]. Together, analyses of two large, high-quality longitudinal datasets explicitly testing whether having children increase pro-environmental engagement do not seem to confirm intuitive predictions of parenthood effects. However, there are still a number of methodological and theoretical considerations to be kept in mind when interpreting the results.

Testing for parenthood effects as outlined in this paper requires a properly-sized longitudinal dataset of sufficient length. While the NZAVS is a high-quality longitudinal dataset with a large sample size (the sample contains over 23,000 unique individuals and more than 78,000 measurement occasions), there were only a limited number of childbirths, in particular of firstborns (of the 1,522 childbirths, around 400 were firstborns). That may not be sufficient to detect what are most likely modest effects. Another consideration is the age of mothers and fathers. The median age of women giving birth to a child is 30 in New Zealand and range between 13 and 53 [35]. The average age fathers is slightly higher (33 years), and around one in 100 babies has a father aged 50 years or over [36]. The average age of our sample is relatively high (i.e., 43.5 years at the time of Wave 1), meaning that many women in the sample are beyond childbearing age.

In addition, while the dataset included multiple waves of data collection and therefore was able to not only detect sudden mean-level but also more gradual slope-level changes before and after childbirth, the analyses were constricted to a six-year period. It is possible that the effect of parenthood on pro-environmental engagement is delayed over a longer period, and that (even) more measurement points are required to detect effects. Environmental attitudes and behaviour following childbirth may also have a U-shaped pattern. Initially, the impact of childbirth on environmental engagement may be negative because of pressures of looking after a young child, which then is followed by an increase in pro-environmental intentions/behaviour to ensure an environmental legacy is left for offspring. Indeed, Thomas et al. [22] observed detrimental effects of having a new-born child in the frequency of three behaviours (i.e., ‘wear more clothes instead of more heating’, ‘use public transport instead of car’ and ‘carshare with others’) that are harder to perform when parenting efforts takes precedent over other concerns. As discussed by Thomas et al. in relation to other findings [12, 23, 37], the pressing concerns of new parents is to dedicate time, resources and energy for the immediate health and wellbeing of offspring, which should outweigh broader and longer-term concerns regarding environmental sustainability. It is possible that parental investment would start to include environmental considerations once the more immediate pressures of parenthood subside, and more measurement points are needed to capture longer-term patterns than were available in this study. Non-linear and delayed effects associated with having are a distinct possibility, as argued here, and should therefore be tested as part of future research using longitudinal datasets of sufficient length.

Major life events that are planned or at least can be anticipated may produce effects in preparation for the event. Indeed, childbirth has been associated with a number of anticipatory psychological and behavioural effects [24, 26, 27, 38]. Anticipatory effects may bias the findings and can be missed with an insufficient number of pre-event measurements. In this study we modelled anticipatory effects in environmental attitudes and self-reported behavioural changes. Limited evidence was found for pre-birth changes, although there was a small but significant negative effect in reported changes in people’s daily environmental routines. This may indicate that possible negative changes in environmental habits may already be initiated in advance of the birth of a child. These anticipatory effects need to be studied in more detail because they may dampen or mask changes that new parents may make in response to the birth of a child.

Another reason for the absence of parenthood effects in this study may be that they only occur in specific groups. For example, Thomas and colleagues [22] found that parents with already high environmental concern show a small increase in the desire to act more sustainably after the birth of their first child. In the current paper we examined possible moderators, such as gender and parenthood status (i.e., whether participants already had a child or not), but there are other socio-demographic, psychological, and situational factors to consider. It is possible that, for example, socio-economic status and (pre-existing) environmental values may moderate potential parenthood effects. Economic circumstances may prevent new parents from making pro-environmental changes, and effects may be the most pronounced for those who are already concerned about the environment, and climate change in particular. Future research could study this in more detail, although other analytical techniques may be needed to study moderation effects as noted below.

In relation to the previous point, parenthood effects on environmentalism is based on the idea that the birth of a child enhances a parent’s legacy motivation. This is a yet untested assumption, mainly because legacy motivation measures have not been available in longitudinal datasets. Previous research has shown that a motivation to leave a positive legacy can be leveraged to increase engagement with climate change and other environmental problems [18], but it is still unclear whether this is also happening in response to having a child. There has been a call to understand environmentally relevant behaviour from a multilevel perspective to examine individual and contextual factors [39], and we extend this call by employing a multilevel analysis to examine changes over the life course. We believe theorising in the field will benefit from datasets that allows examination of developmental trajectories of environmental attitudes and behaviour and how they change as a result of major life events and transitions.

In this study we used a multilevel interrupted time series approach to study abrupt and more gradual changes before and after childbirth. This design is increasingly used in public health intervention [30] and life transition [24] research, as it allows the explicit modelling of the time-dependent nature of outcomes. Our study illustrates the implementation of this analytical strategy in the environmental domain, and previous studies have also used interrupted time series analysis to evaluate intervention outcomes of “natural experiments” with environmental consequences [40, 41]. As with any analytical technique there are limitations. Life transitions are usually associated with a number of changes, and those who experience a transition may be different from those who do not. Parenthood is usually planned in advance, and previous studies have shown that people without children and parents-to-be differ in socio-economic, social, and psychological characteristics (e.g., in personality see [42, 43]). While we were able to control for anticipation effects and for the socio-demographic variables of gender, age, ethnicity and socio-economic deprivation, biases may still occur due to selection effects. Not all participants may have the same propensity to have a child in a particular period, and this may produce or obscure an effect [43, 44].

Different techniques can be used to control for potential selection effects. A propensity score matching approach [44] can be used to match prospective parents with non-parents that have similar baseline characteristics. Balancing characteristics that determine the propensity to experience a specific event or an intervention has become widespread in life transition research to avoid biased treatment effects [5, 24, 27, 45, 46]. It would be necessary to explore propensity effects with further moderation analyses and to increase confidence in the evidence so far that there are no changes in environmental attitudes and behaviour following childbirth.

In conclusion, we examined whether the birth of a new chid increased climate change beliefs and pro-environmental attitudes and behavioural intentions of parents. Overall, our longitudinal analysis shows no mean-level or rate-change effects in the environmental measures examined, disconfirming predictions of parenthood effect on environmentalism. There were no changes observed in either mothers or fathers, similarly disconfirming gender or ‘parental role’ interpretations of possible parenthood effects [1]. While there was a small effect indicating that becoming a parent for the first time may increase beliefs in the reality of climate change, these effects should be considered preliminary given the exploratory nature of those analyses and the fact this becomes statistically non-significant when correcting for multiple comparison. The study contributes to theoretical and methodological advances in environmental decision-making research but should be expanded upon with further analyses to address uncertainties about the specific temporal pattern of effects and potential selection and anticipation effects in becoming a parent. We hope possible parenthood effects on environmentally relevant variables continue to be explored in future studies.

## Supporting information

S1 File (DOCX)
