# Supplementary material for: Does having children increase environmental concern? Testing parenthood effects with longitudinal data from the New Zealand Attitudes and Values Study
Source: PLoS One. 2020 Mar 18;15(3):e0230361. doi: 10.1371/journal.pone.0230361 (PMC7080276; doi:10.1371/journal.pone.0230361)

**Supporting Information**

As detailed in the manuscript, we analysed the data from a multilevel interrupted time series regression perspective using the MLwiN 2.36 software package [1].

Here we report the analyses output from MLwiN for (1) the main analysis as well as the (2) additional analyses examining whether any changes in the dependent variables are evident in both new mothers and new fathers. This was tested by adding an interaction term between participants’ gender and having a new child during the period of the study. Below we detail the variable labels used in the MLwiN output.

**Dependent variables**

**Env.ClimateChgReal** = “Climate change is real”

**Env.ClimateChgCause** = “Climate change is caused by humans”

**Env.SacWilling** = “Are you willing to make sacrifices to your standard of living (e.g., accept higher prices, drive less, conserve energy) in order to protect the environment?”

**Env.SacMade** = “Have you made sacrifices to your standard of living (e.g., accepted higher prices, driven less, conserved energy) in order to protect the environment?”

**Env.RoutineWilling** = “Are you willing to change your daily routine in order to protect the environment?”

**Env.RoutineMade** = “Have you made changes to your daily routine in

order to protect the environment?”

**Independent variables**

**Gender_1** = gender of participants (1=male, 0=female)

**Age.10** = age of participants at the time of the first wave of the study in 2009 (measured in 10 years).

**Ethnicity_1** = ethnicity of participants (1=New Zealand European, 0=other)

**NZDep** = The New Zealand Deprivation Index, ranging from 1 (*least deprived*) to 10 (*most deprived*).

**Year** = survey year (ranging from 0 to 6)

**New.born.new_1** = newborn status, assessing having a new child at the time of the survey (1 = yes, 0 = no).

**New.born.year.new** = newborn year, assessing time elapsed since having the child; measured in years (ranging from 0 to 6).

**Anticipation.year.new** = the time leading up to having a new child; measured in years (ranging from -6 to 0).

**Next.born.parent_1.new.born.new_1** = nextborn status variable, indicating having a new child during the period of the study while already having given birth to, fathered, or adopted a child. This variable is included in the third set of outputs.

**Male.new.born.new_1** = interaction between participants’ gender and having a new child during the period of the study. This variable is included in the second set of outputs

**Reference**

1. Rasbash J, Charlton C, Browne WJ, Healy M, & Cameron B. MLwiN Version 2.36. Bristol: Centre for Multilevel Modelling, University of Bristol; 2016.

**(1) Main results reported in Table 3 of the manuscript (Models 1)**


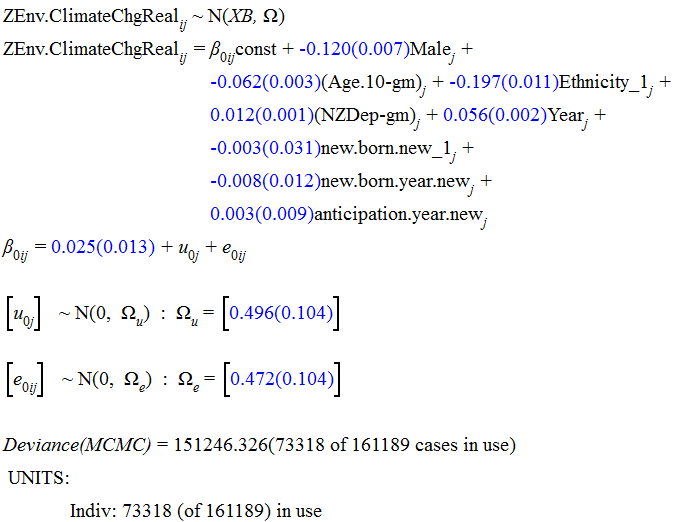

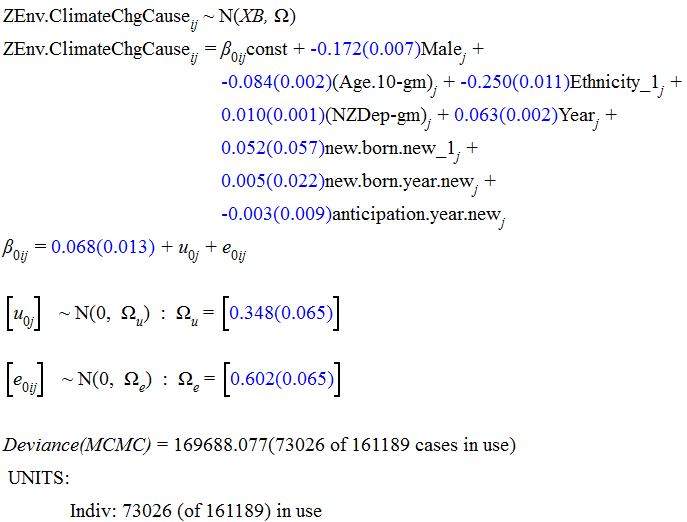


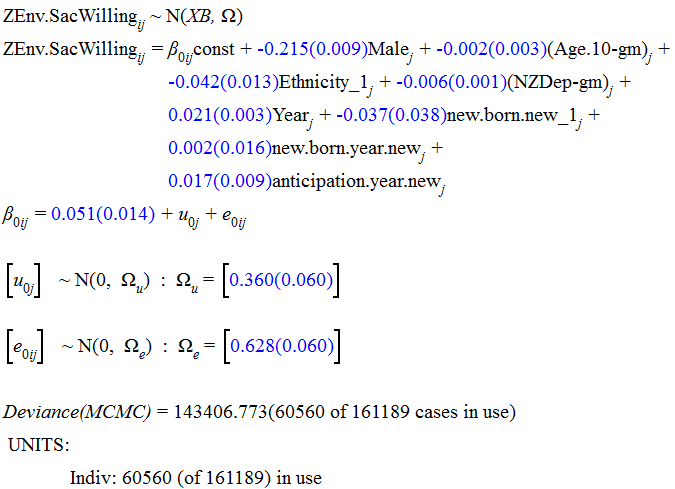


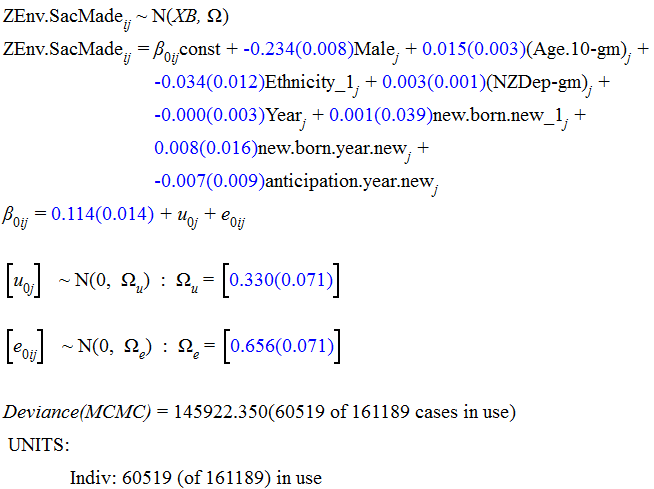


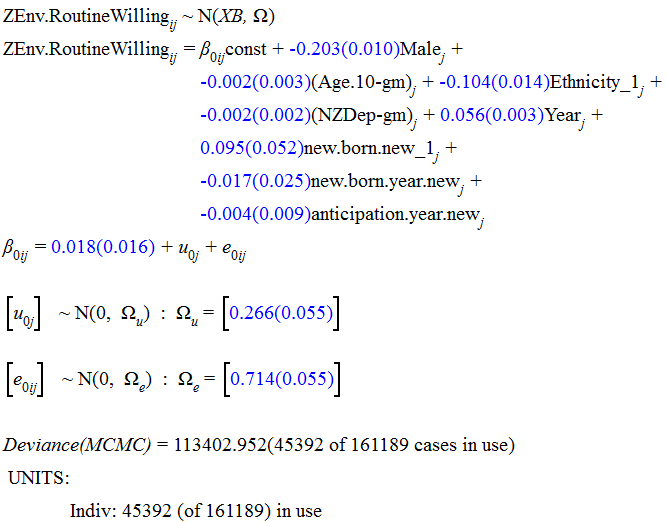


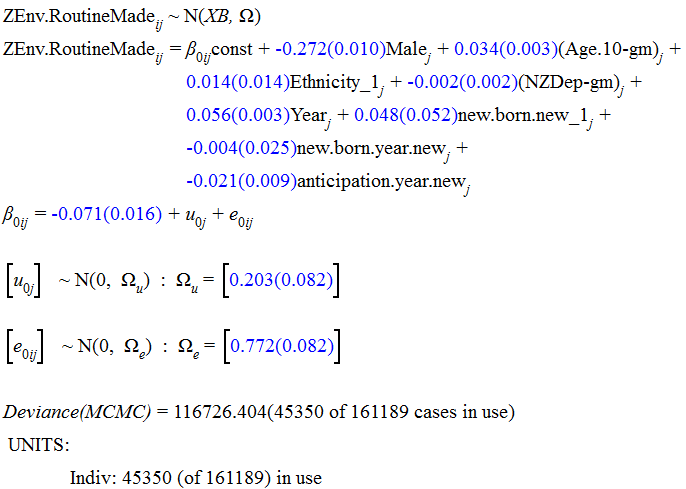


**(2) Main results reported in Table 3 of the manuscript (Models 2 and Models 3)**

Models 2:


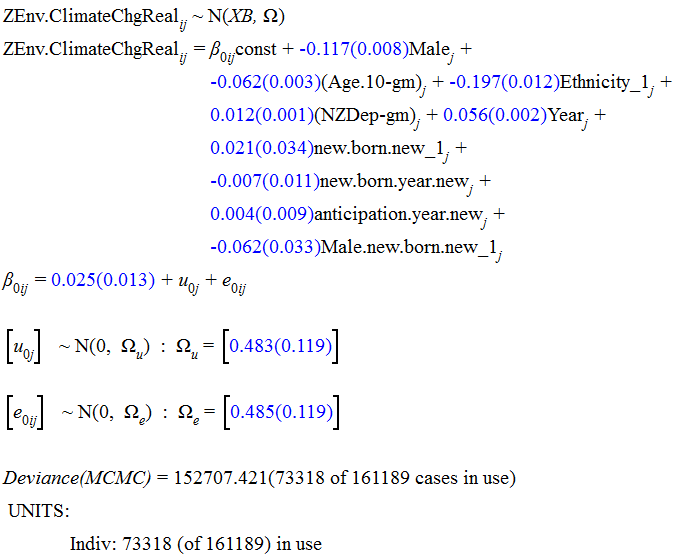


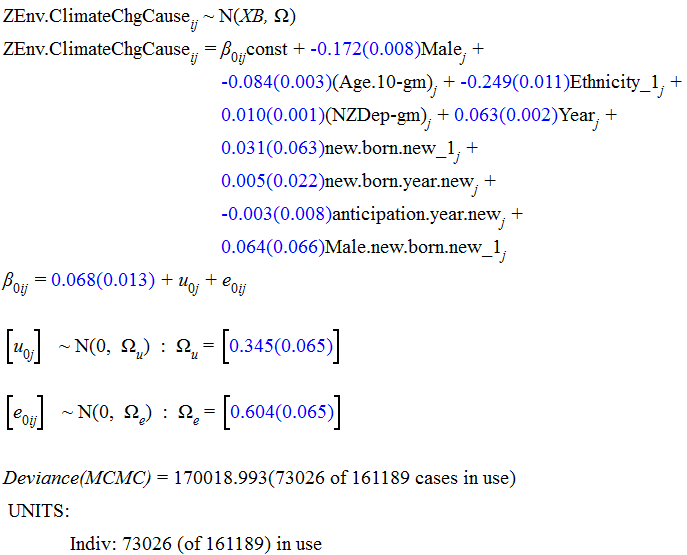


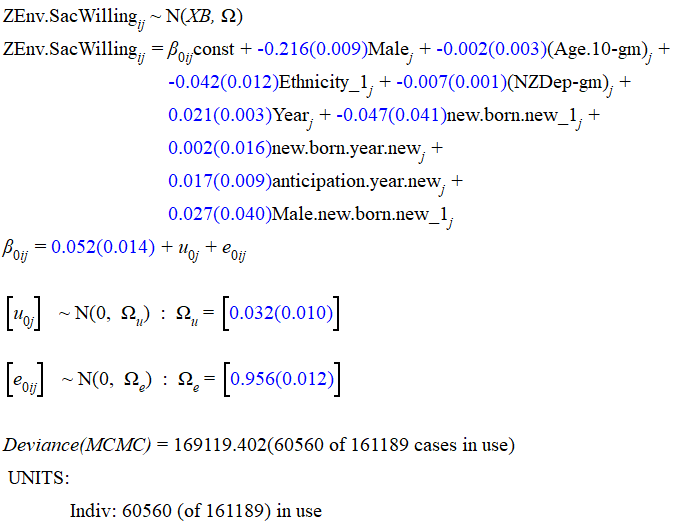


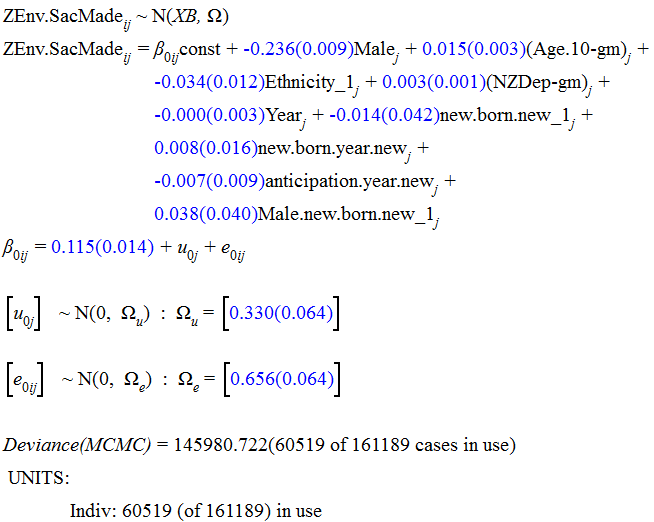


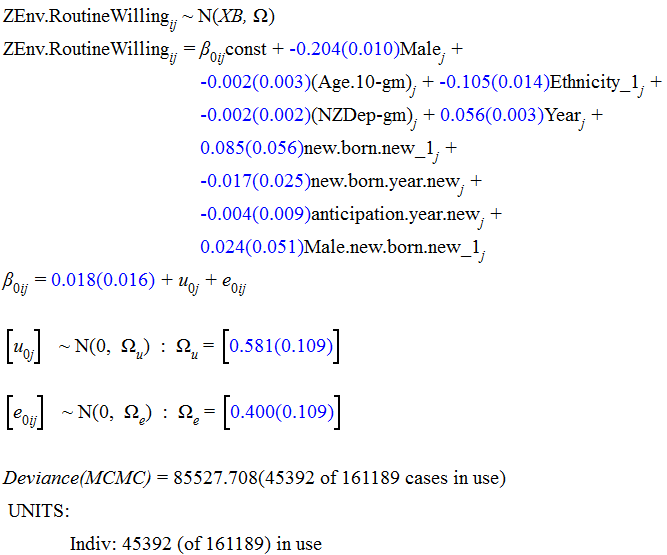


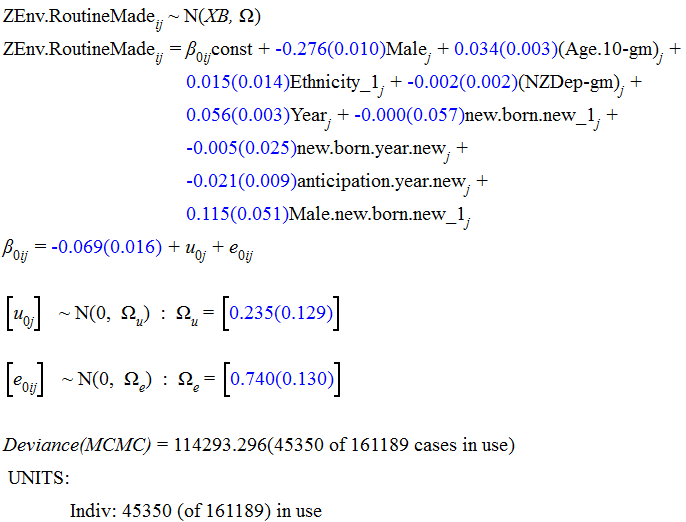


Models 3:


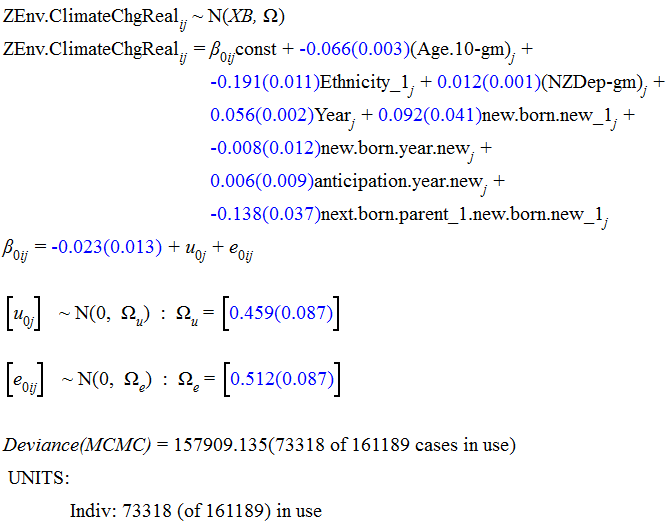


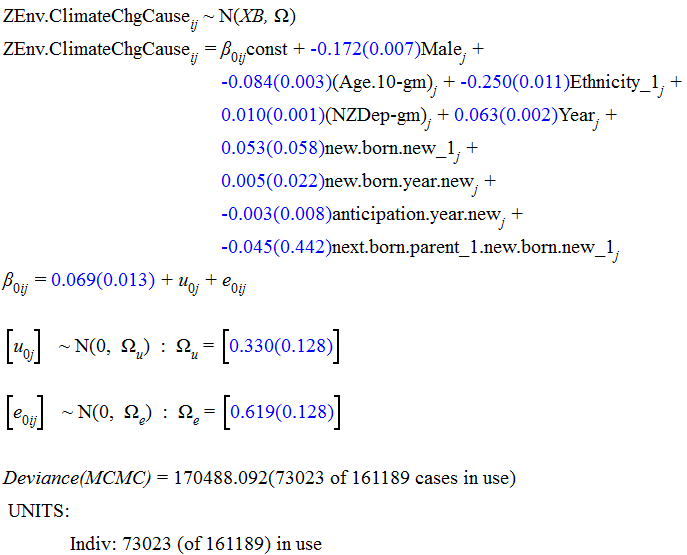


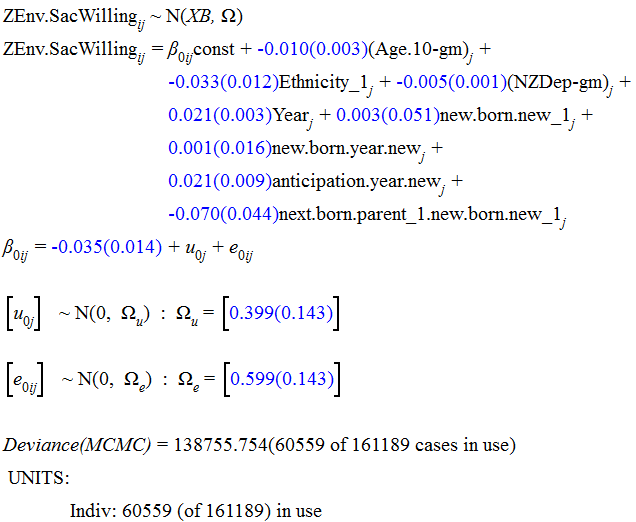


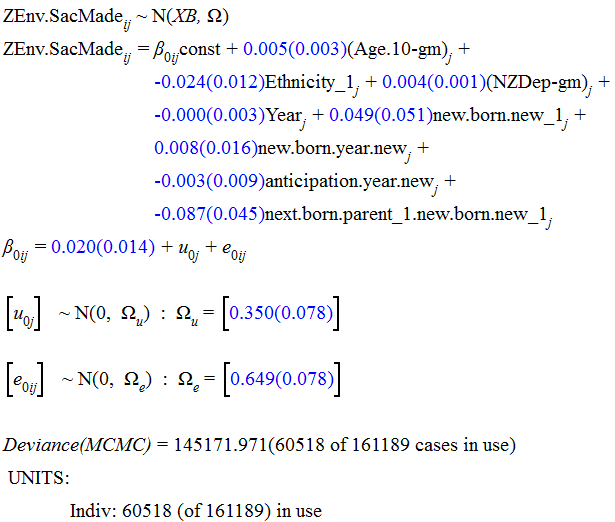


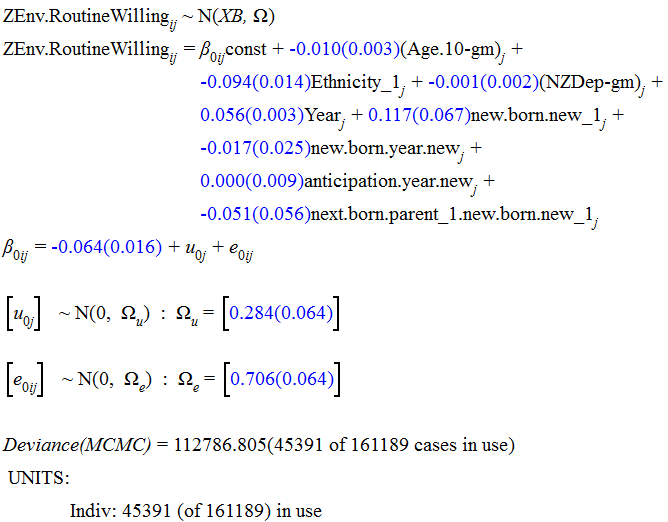


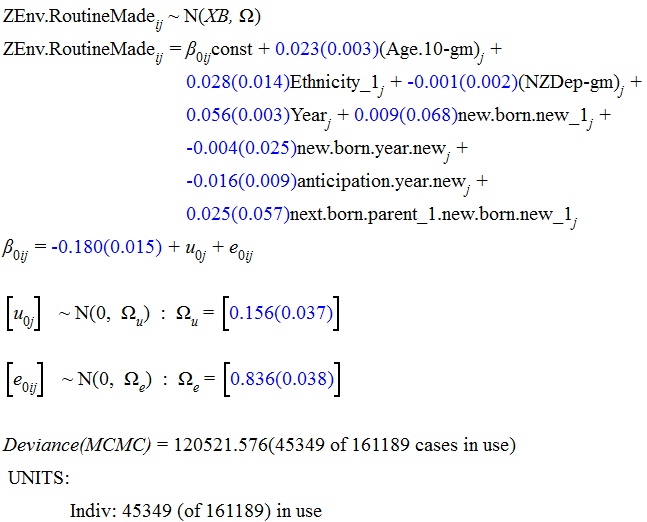

Supplement: S1 File — (DOCX) [file pone.0230361.s001.docx]
